# Within-Mat Variability in Anatoxin-a and Homoanatoxin-a Production among Benthic *Phormidium* (Cyanobacteria) Strains 

**DOI:** 10.3390/toxins4100900

**Published:** 2012-10-19

**Authors:** Susanna A. Wood, Francine M. J. Smith, Mark W. Heath, Thomas Palfroy, Sally Gaw, Roger G. Young, Ken G. Ryan

**Affiliations:** 1 Cawthron Institute, Private Bag 2, Nelson 7042, New Zealand; Email: thomas.palfroy@cawthron.org.nz (T.P.); roger.young@cawthron.org.nz (R.G.Y.); 2 Department of Biological Sciences, University of Waikato, Private Bag 3105, Hamilton 3240, New Zealand; 3 Department of Chemistry, University of Canterbury, Private Bag 4800, Christchurch 8140, New Zealand; Email: Francine.Smith@canterbury.ac.nz (F.M.J.S.); sally.gaw@canterbury.ac.nz (S.G.); 4 School of Biological Sciences, Victoria University of Wellington, PO Box 600, Wellington 6140, New Zealand; Email: Mark.Heath@vuw.ac.nz (M.W.H.); Ken.Ryan@vuw.ac.nz (K.G.R.)

**Keywords:** 16S rRNA gene sequence, *anaF* gene, anatoxin-a, benthic mats, cyanobacteria, homoanatoxin-a, *Phormidium *

## Abstract

Benthic *Phormidium *mats can contain high concentrations of the neurotoxins anatoxin-a and homoanatoxin-a. However, little is known about the co-occurrence of anatoxin-producing and non-anatoxin-producing strains within mats. There is also no data on variation in anatoxin content among toxic genotypes isolated from the same mat. In this study, 30 *Phormidium* strains were isolated from 1 cm^2^ sections of *Phormidium*-dominated mats collected from three different sites. Strains were grown to stationary phase and their anatoxin-a, homoanatoxin-a, dihydroanatoxin-a and dihydrohomoanatoxin-a concentrations determined using liquid chromatography-mass spectrometry. Each strain was characterized using morphological and molecular (16S rRNA gene sequences) techniques. Eighteen strains produced anatoxin-a, dihydroanatoxin-a or homoanatoxin-a. Strains isolated from each mat either all produced toxins, or were a mixture of anatoxin and non-anatoxin-producing genotypes. Based on morphology these genotypes could not be separated. The 16S rRNA gene sequence comparisons showed a difference of at least 17 nucleotides among anatoxin and non-anatoxin-producing strains and these formed two separate sub-clades during phylogenetic analysis. The total anatoxin concentration among toxic strains varied from 2.21 to 211.88 mg kg^−1^ (freeze dried weight), representing a 100 fold variation in toxin content. These data indicate that both the relative abundance of anatoxin and non-anatoxin-producing genotypes, and variations in anatoxin producing capability, can influence the overall toxin concentration of benthic *Phormidium* mat samples.

## 1. Introduction

*Phormidium* is a cyanobacterium that can form expansive benthic mats on the substrata of lakes and rivers [1,2,3]. Benthic proliferations dominated by *Phormidium* are of concern because some strains produce the potent neurotoxic compounds anatoxin-a, dihydroanatoxin-a, homoanatoxin-a, and dihydrohomoanatoxin-a, hereafter collectively referred to as anatoxins [1,2,4]. Anatoxins are powerful neuromuscular blocking agents that act through the nicotinic acetylcholine receptor. They bind to acetylcholine receptors triggering muscle contraction or other neural signal responses. They cannot be broken down by acetylcholine esterase and cause over-stimulation. When ingested, these toxins can cause death via asphyxia from respiratory paralysis [5]. Consumption of anatoxin-containing *Phormidium* mats has resulted in numerous dog poisonings and fatalities worldwide [1,4,6,7]. 

Studies of benthic *Phormidium* mats in rivers across New Zealand have shown a wide variability in anatoxin concentrations [2,4,8,9]. Samples taken from individual sites (100 m^2 ^in area) on the same day can also show a wide divergence in anatoxin content. Both anatoxin and non-anatoxin containing samples were identified and these were sometimes spaced less than 1 m apart [10]. Studies have also noted large differences in toxin concentrations from week to week, and within rivers on the same day [8,9,10].

Cyanobacterial toxin dynamics have been examined in planktonic bloom forming cyanobacteria for many decades with similar extreme variability in toxin concentrations recorded. This variability has been observed spatially and temporally in both long-term and fine scale investigations [11,12]. Within toxic planktonic blooms of anatoxin-a-producing *Aphanizomenon issatschenkoi * [13], saxitoxin-producing *Anabaena* [14], and microcystin-producing *Microcystis * [15,16], or *Planktothrix *[17], it has now been demonstrated that this variability is largely due to the co-occurrence of toxic and nontoxic genotypes. Differences in toxin content can be explained by the relative dominance of toxin-producing or non-producing strains. Culture based studies of strains isolated from blooms have also shown that toxic genotypes vary in the amounts of toxin and specific congeners produced [14,18]. Despite an increasing number of reports of toxic benthic cyanobacteria [19,20,21,22,23] there is only very limited information available on the co-occurrence of toxic and non-toxic strains [2,10,24]. There is no information on variation in anatoxin content among toxic strains isolated from the same benthic mat.

In this study we investigated the toxin producing abilities of 30 *Phormidium* strains isolated from four anatoxin-containing mats collected from three different sites. The anatoxin production potential of each strain was assessed using a PCR targeting the *anaF *gene. Strains were grown to stationary phase and their anatoxin content determined using liquid chromatography-mass spectrometry (LC-MS). Each strain was characterized using morphological and molecular (16S rRNA gene sequences) techniques.

## 2. Results

A total of 30 *Phormidium* strains were established from single filaments. Ten strains (CYN103–CYN112) were isolated from the Waimakariri River mat, four (CYN113, CYN115–117) and eight strains (CYN126–133) were isolated from mat 1 and mat 2 from Hutt River, Site 1 respectively, and eight strains (CYN118–125) were isolated from a mat collected at Hutt River, Site 2. 

Anatoxin-a and dihydroanatoxin-a were detected in 18 of the 30 samples. One of these strains (CYN112), isolated from the Waimakariri River, also produced homoanatoxin-a (Table 1). All of the strains isolated from mat 1 from the Hutt River, Site 1, produced anatoxins. In contrast not all of the isolates from the other mats produced anatoxins; nine out of ten isolated from the Waimakariri River, one out of eight from the Hutt River, Site 2, and six out of eight from mat 2 from the Hutt River, Site 1 did not contain anatoxins (Table 1).

Among the anatoxin producing strains isolated, the concentration of anatoxins produced varied and there was no relationship between the concentration produced and the original source. For example, total anatoxins produced by the isolates from the Waimakariri River varied from 10.06 to 211.83 mg kg^−1^ and from 4.75 to 115.42 mg kg^−1 ^dry weight for isolates from mat 1, Hutt River, Site 1. Dihydroanatoxin-a concentrations were always higher than anatoxin-a (Table 1).

All strains that were found to produce anatoxins via LC-MS also tested positive by PCR for a segment of the *anaF* gene (Table 1). Gene segments (404 bp) from strains CYN103–107 and 109–112 were sequenced and were identical. Using Megablast, these segments shared 99% sequence homogeneity with an *anaF* gene segment from strain CYN53 (JX088093), a *P. autumnale* strain isolated from the Rangitaiki River (North Island, New Zealand; [2]). The next closest sequence similarities were 94% with *Oscillatoria* PCC 6506 (FJ477836), and 91% with *Anabaena* sp. (JF803645) and *A. flos-aquae *(AY210784). 

**Figure 1:**
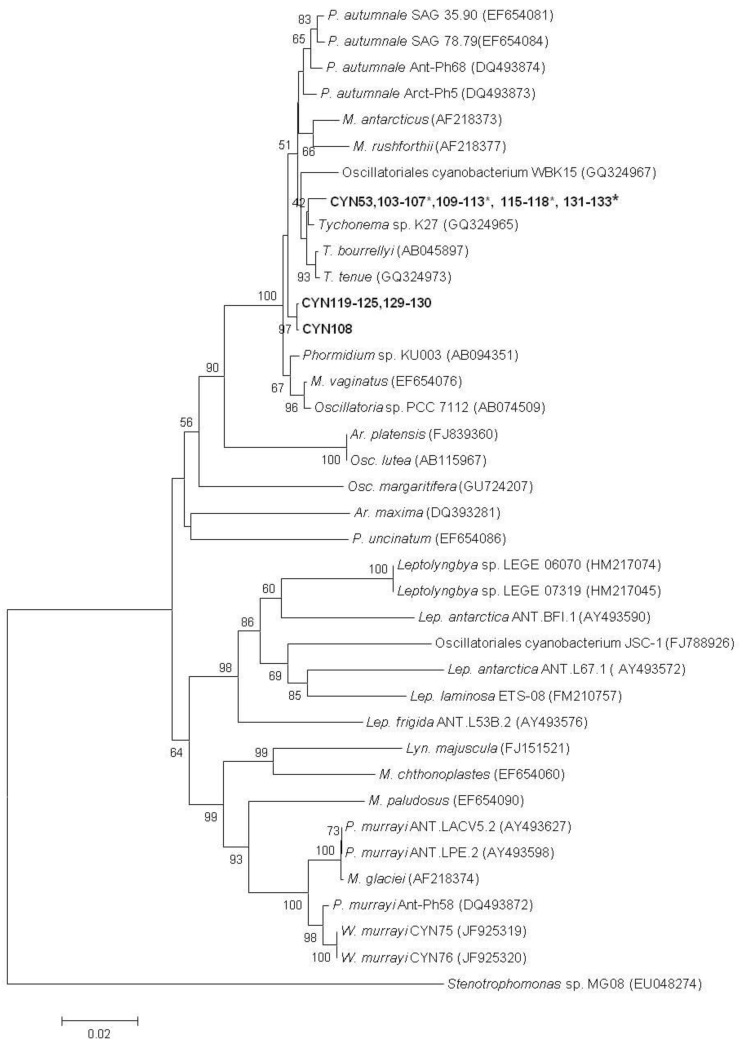
Phylogenetic tree based on 16S rRNA gene sequences (1340 bp). The tree was constructed using the neighbour-joining method. Bootstrap values >50% are noted at the nodes.Scale bar = 0.02 substitutions per site. *Ar = Arthrospira*, Lep = *Leptolyngbya*, *Lyn = Lyngbya*, *M *= *Microcoleus*, *Osc* = *Oscillatoria*, *P* = *Phormidium*, *T* = *Tychonema*, *W = Wilmottia*.* anatoxin-producing strains isolated in this study.

Partial 16S rRNA gene sequences (1340 bp) were obtained for all strains. The strains which tested positive for anatoxins (CYN103–107, 109–113, 115–118, 126–128 and 131-133) were identical over this region (Figure 1). These strains were also identical to CYN53 (JX088083). These segments had a 99% sequence homology with *Tychonema* sp. K27 (GQ324965), *T. bourrellyi* CCAP (AB045897) and *T. tenue* SAG 4.82 (GQ324973). The sequences from all other strains isolated in this study, except CYN108, were identical, and were 17 nucleotides different from the anatoxin positive strains. When submitted to Megablast the closest matches (99%) for these strains were: *Phormidium* sp. KU003 (AB094351), *Microcoleus vaginatus* SEV1-KK3, CJI-U2-KK1, PCC 9802 (EF654076, EF654078, AF284803), *P. autumnale* SAG 35.90, Ant-Ph68, Arct-Ph5 16S, SAG 78.79 (EF654081, DQ493874, DQ493873, EF654084), *Tychonema* sp. K27 (GQ324965), *T. bourrellyi* CCAP (AB045897) and *T. tenue* SAG 4.82 (GQ324973). Strain CYN108 differed from the other anatoxin negative strains by four nucleotides and an insertion of 10 nucleotides. Megablast analysis returned the same results as obtained for the other anatoxin negative strains.

**Table 1:** Results of the *anaF* PCR and liquid chromatography-mass spectrometry (LC-MS) of 30 strains of *Phormidium* isolated from the Waimakariri (WR) and Hutt (HR) rivers. -1-1 = Site 1, mat 1, -2 = Site 2, -1-2 = Site 1, mat 2, ND = Not detected, ATX = anatoxin-a, dhATX = dihydroanatoxin, HTX = homoanatoxin-a, dhHTX = dihydrohomoanatoxin. Concentrations of dhATX, HTX and dhHTX were calculated using the anatoxin-a calibration curve.

Strain	Location	*anaF* PCR	LC-MS data (mg kg^−1^)				
			ATX	dhATX	HTX	dhHTX	TOTAL
CYN103	WR	+	5.92	205.92	ND	ND	211.83
CYN104	WR	+	5.79	156.07	ND	ND	161.86
CYN105	WR	+	0.28	40.80	ND	ND	41.08
CYN106	WR	+	0.47	56.39	ND	ND	56.86
CYN107	WR	+	0.93	9.13	ND	ND	10.06
CYN108	WR	-	ND	ND	ND	ND	ND
CYN109	WR	+	3.25	128.38	ND	ND	131.63
CYN110	WR	+	6.40	171.80	ND	ND	178.20
CYN111	WR	+	1.08	71.90	ND	ND	72.98
CYN112	WR	+	2.38	165.75	1.00	ND	169.13
CYN113	HR-1-1	+	0.04	66.91	ND	ND	66.95
CYN115	HR-1-1	+	0.25	28.57	ND	ND	28.83
CYN116	HR-1-1	+	0.04	31.29	ND	ND	31.33
CYN117	HR-1-1	+	0.12	152.16	ND	ND	152.28
CYN118	HR-2	+	0.02	2.19	ND	ND	2.21
CYN119	HR-2	-	ND	ND	ND	ND	ND
CYN120	HR-2	-	ND	ND	ND	ND	ND
CYN121	HR-2	-	ND	ND	ND	ND	ND
CYN122	HR-2	-	ND	ND	ND	ND	ND
CYN123	HR-2	-	ND	ND	ND	ND	ND
CYN124	HR-2	-	ND	ND	ND	ND	ND
CYN125	HR-2	-	ND	ND	ND	ND	ND
CYN126	HR-1-2	+	0.19	115.23	ND	ND	115.42
CYN127	HR-1-2	+	0.63	79.92	ND	ND	80.55
CYN128	HR-1-2	+	0.12	32.58	ND	ND	32.70
CYN129	HR-1-2	-	ND	ND	ND	ND	ND
CYN130	HR-1-2	-	ND	ND	ND	ND	ND
CYN131	HR-1-2	+	ND	4.75	ND	ND	4.75
CYN132	HR-1-2	+	0.25	74.32	ND	ND	74.58
CYN133	HR-1-2	+	0.06	73.56	ND	ND	73.62

The 16S rRNA gene sequences were used to construct a phylogenetic tree. The anatoxin and non-anatoxin producing strains isolated in this study clustered in a single clade with 100% bootstrap support (Figure 1). The anatoxin-producing strains clustered most closely with *Tychonema* species, whilst the non-toxic strains (CYN108, 119–125, 129, 130) formed their own sub-clade (Figure 1).

All strains shared a similar morphology although there was considerable heterogeneity among cell lengths and widths. Filaments consisted of a single trichome within a firm colourless sheath. Trichomes were light to dark brown, motile, gradually attenuated towards apical cells, commonly forming hormogonia and not constricted at cross-walls. Cells were generally shorter than wide with some cells having densely arranged granules at cross-walls. Apical cells were rounded and occasionally capitate or calyptrate when mature. Cell dimensions among anatoxin producing strains (CYN103–107, 109–113, 115–118, 126–128 and 131–133) were 5.8–12.5 μm wide by 1.2–7.8 μm long. Among the non-anatoxin-producing strains (CYN108, 119–125, 129–130) the cell dimensions were 6.4–10.6 μm wide by 2.4–4.8 μm long.

## 3. Discussion

The results of this study demonstrate, for the first time, the coexistence of anatoxin- and non-anatoxin-producing *Phormidium * strains within small (1 cm^2^) regions of benthic mats. This co-occurrence of anatoxin and non-anatoxin-producing genotypes within benthic mats has been postulated previously, but not proved conclusively. Heath *et al.*, [2] showed evidence for their co-occurrence by isolating seven strains of *P. autumnale *from mats that tested positive for anatoxins using LC-MS. Five of the seven strains did not produce anatoxins. Similarly, anatoxin- and non-anatoxin-producing strains of *Phormidium *were isolated from floating scum samples from one site on the River Tarn, France; however these strains were isolated from samples collected on different dates [24]. Despite the notably different habitats and species involved, the data from this study concurs with planktonic cyanobacteria studies where the co-occurrence of toxic and non-toxic genotypes is now well documented [13,14,15,16,17]. Molecular studies on natural populations of planktonic cyanobacteria have shown that the proportion of toxic and non-toxic genotypes varies as blooms progress. In many instances, these shifts in genotype composition correlate with changes in toxin concentration [25]. Although PCR assays targeting a range of genes involved in the biosynthesis of anatoxin have been developed [26], the use of quantitative PCR (QPCR) to assess changes in genotype abundance has not yet been applied to the study of benthic *Phormidium*. To determine if there is a relationship between the relative abundances of anatoxin and non-anatoxin-producing benthic *Phormidium* and anatoxin concentrations, QPCR assays targeting genes involved in anatoxin biosynthesis need to be used to study environmental sample sets.

The total anatoxins concentration of the strains isolated in this study varied from 2.21 to 211.83 mg kg^−1^ (freeze dried weight). This represents a 100 fold variation in toxin content among toxic strains. Thus in addition to the relative abundance of anatoxin and non-anatoxin-producing genotypes, variations in toxin producing capability among toxic genotypes can influence the overall toxin concentration of a benthic mat sample. The differences in anatoxin production among strains are not apparent from the presence or absence of *anaF* or other anatoxin biosynthetic genes. These data highlight the utility of isolating strains and using chemical analysis to increase knowledge on variations in toxin production. Anatoxin concentrations from this study (maximum 211.83 mg kg^−1^), were markedly lower than those measured for *P. autumnale* strain CYN53 (Rangitaiki River, North Island, New Zealand; [2]), which consistently produces approximately 1000 mg kg^−1^ of anatoxins [2]. This finding demonstrates that the difference between the most and least toxic strains is even greater than measured in this study. Long-term culturing of cyanobacteria can result in changes in toxin production [27]. In this study, all strains were isolated at the same time and maintained under identical culture conditions and therefore the differences in the quantitative amounts of anatoxins are likely to represent significant differences between strains.

A further important consideration is the relative abundances of the different anatoxin congeners and the effect of this on toxicity. Anatoxin-a and homoanatoxin-a have a mouse LD_50_ (lethal dose resulting in 50 percent deaths) of 200–250 µg kg^−1^ body weight [28,29]. Dihydroanatoxin-a has an LD_50_ of 2 mg kg^−1^ [30]. Among the strains in this study that contained both anatoxin-a and dihydroanatoxin-a, there was between 10 to 1600 times more dihydroanatoxin-a produced (Table 1). In contrast CYN53 produces predominately anatoxin-a [2], thus as well as producing high levels of anatoxin-a it is also producing the congener variant with the highest toxicity. 

All of the strains isolated in this study fell into a single clade supported with 100% bootstrap support within the well-defined 16S rRNA gene sequence *P. autumnale *clade (Figure 1). The *P. autumnale * clade has recently been confirmed by morphological and ultrastructure data [31,32]. In the current study all of the strains shared close homology (>99%) with species of *Microcoleus *and *Tychonema*. This observation has previously been observed for 16S rRNA sequences of *P. autumnale *strains [2,31,32]. Previous studies on planktonic cyanobacteria have shown that there is no, or very limited, difference in 16S rRNA gene sequences between toxic and non-toxic strains [33,34]. In contrast, in this study the anatoxin and non-anatoxin-producing strains clearly fell into two separate sub-clades. Based on analysis of a 1340 base pair region of the 16S rRNA gene sequence, there was at least a 17 nucleotide differences between the genotypes. Cadel-Six [24] studied benthic cyanobacteria using a different phylogenetic marker and also observed similar results. They showed that the internal transcribed spacer regions (ITS), ITS1 and ITS2, and the 5.8S regions of rRNA operon could provide sufficient resolution to demonstrate that each neurotoxic phenotype corresponded to a distinct genotype. Based on the observed differences in the 16S rRNA gene sequences it may be possible to design a PCR-based assay to separate these genotypes. However, for distinguishing toxic and non-toxic genotypes, PCR-based assays targeting genes involved in anatoxin biosynthesis [26] would be more conclusive in identifying anatoxin potential than using an assay based on 16S rRNA gene sequences. 

It is well known from research on planktonic cyanobacteria that morphology alone cannot be used to distinguish toxic and non-toxic genotypes [35,36]. A similar result has been shown for some benthic species [2] and despite variability in the 16S rRNA gene sequence phylogeny, difference in morphology could not be used to separate anatoxin and non-anatoxin-producing genotypes. This finding is consistent with Comte *et al.*, [31] who were unable to separate *P. autumnale* strains Arct-Ph5 and Ant-Ph68 based on morphological characters, but identified them as two subspecies by genetic analysis (16S rRNA gene sequences). The general morphology of all strains in this study was similar and there was considerable variability in cell dimension within both toxic and non-toxic strains. *Phormidium autumnale * displays considerable plasticity, with strains varying from 1.6 to 13 μm wide by 1 to 13 μm long [37]. 

## 4. Methods

### 4.1. Sample Sites and Collection

Samples were collected from three sites during benthic proliferation events; Waimakariri River (South Island, New Zealand, 43°25'09"S 172°38'01"E, April 2011), two separate mats from the Hutt River-Site 1, North Island, New Zealand, 41°11'19"S 174°56'08"E, January 2008 and one mat from the Hutt River-Site 2, North Island, New Zealand, 41°11'19"S 174°56'08"E, January 2008. At each site the sample was collected by scraping a 1 cm^2^ area of *Phormidium* mat from one rock into a sterile Falcon tube (50 mL). Samples were kept chilled during transport and then frozen (−20 °C) for culturing at a later date. 

### 4.2. Cyanobacterial Culture Isolation

Frozen benthic mat samples were thawed and a subsample (*ca*. 20 mg) from each sample streaked on to MLA agar (1% *w*/*v*, [38]). Half of the Petri dish containing the streaked material was covered with a black polyvinyl carbonate bag to block light. After 2–3 weeks, single filaments that had migrated across the solid MLA were isolated by micro-pipetting, or by cutting around the agar of a single filament. Isolated trichomes were transferred to 24-well plates containing 500 µL of liquid MLA medium per well. Filaments were washed with MLA and incubated under standard conditions (80 ± 15 µmol photons m^−2^.s^−1^; 12:12 h light: dark; 18 ± 1 °C). Successfully isolated strains were maintained in 50-mL plastic bottles (Biolab, New Zealand) under the conditions above. All cultures have been cryopreserved and banked in the Cawthron Institute Culture Collection of Micro-algae (Nelson, New Zealand).

### 4.3. Anatoxin-a and Homoanatoxin-a Extraction and Detection

All cyanobacterial cultures were grown until the early stationary phase. At this phase the cultures had detached from the culture bottle and formed cohesive surface mats which were removed using sterile forceps. Cultures were lyophilized and a sub-sample (100 mg) resuspended in 10 mL of MilliQ water containing 0.1% formic acid. Samples were sonicated (15 min) and centrifuged (4000 × *g*, 10 min). An aliquot of the supernatant was analysed directly for the anatoxins using LC-MS as described in Heath *et al.* [2,4]. There are no commercially available standards for dihydroanatoxin-a, homoanatoxin-a, dihydrohomoanatoxin-a and concentrations were calculated using the anatoxin-a calibration curve.

### 4.4. Morphological and Molecular Analysis

Cyanobacterial cultures were identified using an Olympus light microscope (BX51) at 400–2000× magnification with reference to descriptions in Komárek and Anagnostidis [39] and McGregor [40]. Morphological features were recorded including length and width of vegetative cells (*n* = 30), granulation, and apical cell shape.

Molecular analysis of a segment of the 16S rRNA gene sequence was used to characterise the strains and each was screened for a region of *anaF*, a gene involved in the biosynthesis of anatoxin-a [41]. Subsamples (*ca*. 1 mL) from each strain were centrifuged (10,000 × *g*, 1 min) and the supernatant removed by sterile pipetting. DNA was extracted from the pellets using the PureLinkTM Genomic DNA Kit (Invitrogen, Carlsbad, CA, USA) according to the gram-negative bacteria protocol supplied by the manufacturer. PCR amplification of a region of the *anaF* and segments from the 16S rRNA gene were performed in a three separate 200 μL reaction tubes containing 10 μL of i-Taq 2 × PCR master mix (Intron, Gyeonggi-do, Korea), 1.2 μg non-acetylated bovine serum albumin (Sigma-Aldrich, St. Louis, MO, USA), 0.4 μM of each primers (either atxoaf/atxar [13] for the *anaF* gene, or 27F/809R [42] and 740F/1494R [34,43] for the 16S rRNA gene), and approximately 30 ng of template DNA. Thermal cycling conditions for the *anaF* PCR were an initial denaturation at 94 °C for 4 min; followed by 35 cycles of denaturation at 94 °C for 30 s, annealing at 55 °C for 30 s and extension at 72 °C for 1 min; then a final extension at 72 °C for 7 min. Conditions for the two 16S rRNA PCR’s were an initial denaturation at 94 °C for 2 min; followed by 94 °C for 1 min, 54 °C for 1 min, 72 °C for 2 min, repeated for 30 cycles; with a final extension at 72 °C for 7 min. PCR was undertaken on a DNA Engine thermal cycler (Biorad, Hercules, CA, USA). PCR products were visualized by 1% agarose gel electrophoresis with ethidium bromide staining and UV illumination. Amplicons of the correct size were purified using AxyPrep PCR cleanup kits (Axygen, Union City, CA, USA). Sequencing of selected *anaF* amplicons and all 16S rRNA amplicons was undertaken using the primers given above and the 3730 × l DNA Analyser (Applied Biosystems, Carlsbad, CA, USA) using the BigDye Terminator v3.1 Cycle Sequencing Kit (Applied Biosystems, Carlsbad, CA, USA). Sequences generated during this work were compared with sequences from the NCBI GenBank database using Megablast [44] and then deposited in GenBank under accession numbers JX847017-JX847036 and JX088073-JX088093. All sequences were trimmed and aligned using ClustalW in MEGA 4 [45]. A phylogenetic tree was constructed using a neighbor-joining algorithm [46] and Tamura-Nei distance estimates. Pairwise distances were calculated using the Jukes-Cantor method and pairwise deletion was used to account for sequence-length variation and gaps. Bootstrap analyses of 1000 iterations were performed to identify the node support for consensus trees.

## 5. Conclusions

Spatial and temporal variability in anatoxin concentration occurs among benthic *Phormidium* mats [2,8,9]. In this study, 30 strains of *Phormidium* were isolated from four small sections of benthic mats. Both anatoxin and non-anatoxin-producing genotypes were found to co-occur. There were also 100 fold differences in the amount of anatoxin produced by the toxic strains isolated from a single mat. We speculate that the ratio of anatoxin to non-anatoxin-producing strains, and the varying toxin producing capabilities of each toxic strain within a benthic mat are responsible for the dramatic shifts in anatoxin concentration observed in our previous studies (for example, from 558 to 35 mg kg^−1^ over 7 days [47]). In-depth studies on environmental samples using quantitative molecular techniques will be required to confirm these hypotheses.
